# Fault diagnosis of gearbox based on Fourier Bessel EWT and manifold regularization ELM

**DOI:** 10.1038/s41598-023-40369-1

**Published:** 2023-09-02

**Authors:** Ke Wang, Fengqing Qin

**Affiliations:** Faculty of Artificial Intelligence and Big Data, Yibin University, Yibin, 644000 China

**Keywords:** Engineering, Mathematics and computing

## Abstract

The novel fault diagnosis method of gearbox based on Fourier Bessel series expansion-based empirical wavelet transform (FBEWT) and manifold regularization extreme learning machine (MRELM) is proposed to obtain excellent fault diagnosis results of gearbox in this paper. A new feature extraction strategy based on Fourier Bessel series expansion-based empirical wavelet transform is used to capture the key non-stationary features of the vibrational signal of gearbox, and significantly improve the diagnosis ability of gearbox. The ELM with manifold regularization is proposed for fault diagnosis of gearbox. In order to outstand the superiority and stability of the proposed FBEWT and manifold regularization ELM, the balanced dataset and unbalanced dataset, respectively, are used. The experimental results testify that FBEWT-MRELM are more superior and stable than FBEWT-ELM, EWT-MRELM, and EWT-ELM regardless of balanced dataset and unbalanced dataset.

## Introduction

Gearbox is widely used as a common mechanical transmission component in machines and used in industry to transmit torque and power^1–4^. Due to its harsh working environment, the gearbox may have various faults, such as wear and tear, snaggletooth and tooth pitting. Recently, lots of scholars have studied fault diagnosis methods of gearbox. For instance, Lorenzo proposed a new three-stage gearbox concept for high reduction ratios: use of a nested-cycloidal architecture to increase the power density^5^. Tomasz proposed a tram gearbox condition monitoring method based on trackside acoustic measurement^6^. Hemanth proposed a component level signal segmentation method for multi-component fault detection in a wind turbine gearbox^7^.

It is well-known that a superior feature extraction strategy is key to obtaining excellent fault diagnosis results^8–10^. Empirical wavelet transform is a new adaptive signal decomposition method that inherits the respective advantages of EMD and wavelet analysis methods^11,12^. It adaptively segments the Fourier spectrum by extracting maximum points in the frequency domain to separate different modes. However, empirical wavelet transform segments many invalid components and exists modal aliasing. FBEWT method is a combination of two signal processing technologies: FB and EWT, which is used to analyze non-stationary signals, and Fourier Bessel based on Bessel function is suitable for analyzing time series signals. Therefore, a new feature extraction strategy based on Fourier Bessel series expansion-based empirical wavelet transform is used to capture the key non-stationary features of the vibrational signal of gearbox, and significantly improve the diagnosis ability of gearbox. FBEWT of the vibrational signal of gearbox generates Fourier Bessel intrinsic mode functions (FBIMF).

Extreme learning machine is an efficient single-hidden layer feed-forward network^13^, which has widely applied in the field of fault diagnosis. For instance, Francisco proposed the cascade feature selection and ELM for automatic fault diagnosis of the Tennessee Eastman process^14^. Most of the existing deep limit learning machines use AE-ELM as the basic module to build a multi-layer network structure to achieve the abstract extraction of data sample features. However, AE-ELM are easily trapped in local minima. Therefore, the ELM with manifold regularization is proposed for fault diagnosis of gearbox, which can better maintain the local manifold structure of data in multi-layer unsupervised learning.

The novel fault diagnosis method of gearbox based on FBEWT and manifold regularization ELM is proposed to obtain excellent fault diagnosis results of gearbox in this paper. In order to outstand the superiority and stability of the proposed FBEWT and manifold regularization ELM, the balanced dataset and unbalanced dataset, respectively, are used. The experimental results testify that FBEWT-MRELM are more superior and stable than FBEWT-ELM, EWT-MRELM, and EWT-ELM regardless of balanced dataset and unbalanced dataset.

## Fourier Bessel series expansion-based EWT

Empirical wavelet transform is a method to adaptively extract different modes of non-stationary signals by constructing adaptive wavelets^15–18^. In the empirical wavelet transform, the selection of parameter $\sigma$ makes a very small overlap between two subsequent frequency components. The empirical scaling function and empirical wavelet are expressed by Eqs. (1) and (2), respectively:

1 $$\tau_{n} (\omega ) = \left\{ {\begin{array}{*{20}c} 1 & {\left| \omega \right| \le \left( {1 - \sigma } \right)\omega_{n} } \\ {cos\left[ {\frac{\pi }{2}\beta \left( {\frac{1}{{2\sigma \omega_{n} }}\left( {\left| \omega \right| - \omega_{n} + \sigma \omega_{n} } \right)} \right)} \right]} & {\left( {1 - \sigma } \right)\omega_{n} \le \left| \omega \right| \le \left( {1 + \sigma } \right)\omega_{n} } \\ 0 & {otherwise} \\ \end{array} } \right.$$

2 $$\psi_{n} (\omega ) = \left\{ {\begin{array}{*{20}c} 1 & {\left( {1 + \sigma } \right)\omega_{n} \le \left| \omega \right| \le \left( {1 + \sigma } \right)\omega_{n + 1} } \\ {\cos \left[ {\frac{\pi }{2}\beta \left( {\frac{1}{{2\sigma \omega_{n + 1} }}\left| \omega \right| - \omega_{n + 1} + \sigma \omega_{n + 1} } \right)} \right]} & {\left( {1 - \sigma } \right)\omega_{n + 1} \le \left| \omega \right| \le \left( {1 - \sigma } \right)\omega_{n + 1} } \\ {\sin \left[ {\frac{\pi }{2}\beta \left( {\frac{1}{{2\sigma \omega_{n} }}\left| \omega \right| - \omega_{n} + \sigma \omega_{n} } \right)} \right]} & {\left( {1 - \sigma } \right)\omega_{n} \le \left| \omega \right| \le \left( {1 - \sigma } \right)\omega_{n} } \\ 0 & {otherwise} \\ \end{array} } \right.$$

The approximation coefficients $W_{f}^{\varepsilon } \left( {0,t} \right)$ are obtained by the inner products of the signal and the scaling function and described by the following formula:

3 $$W_{f}^{\varepsilon } \left( {0,t} \right) = \left\langle {y,\tau_{n} } \right\rangle = IFFT\left( {y(x)\tau_{n} (\omega )} \right)$$

The detailed coefficients $W_{f}^{\varepsilon } \left( {n,t} \right)$ are obtained by the inner product of the signal and the empirical wavelets and expressed by the following formula:

4 $$W_{f}^{\varepsilon } \left( {n,t} \right) = \left\langle {y,\psi_{n} } \right\rangle = {\text{IFFT}}\left( {y(x)\psi_{n} (\omega )} \right)$$

Finally, the constructed signal based on EWT is obtained by the following formula:

5 $$f\left( t \right) = {\text{IFT}}\left( {{\text{FT}}\left( {W_{f}^{\varepsilon } \left( {0,t} \right) \times \tau_{n} \left( t \right) + \sum\limits_{n = 1}^{N} {W_{f}^{\varepsilon } \left( {n,t} \right)} \times \psi_{n} \left( t \right)} \right)} \right)$$

FBEWT method is a combination of two signal processing technologies: FB and EWT, which is used to analyze non-stationary signals. It is found that Fourier Bessel based on Bessel function is suitable for analyzing time series signals. EWT works on adaptive wavelet filter banks. These constructed wavelet filters help to segment the FB spectrum. In this study, the FBEWT method is used to extract narrow subband signals from vibration signals of gearbox. Then, empirical scaling and wavelet functions are applied to design band-pass filters on each adaptive segment of the FB spectrum. Based on the concept of Paley and Meyer wavelet, a wavelet based band-pass filter is constructed. The mathematical equations of an empirical scaling function $\tau_{v} (\omega )$ and wavelet function $\psi_{v} (\omega )$ of EWT are expressed by the following formula:

6 $$\tau_{v} (\omega ) = \left\{ {\begin{array}{*{20}c} 1 & {\left| \omega \right| \le \left( {1 - u} \right)\omega_{v} } \\ {cos\left[ {\frac{\pi }{2} \times \Gamma \left( {u,\omega_{v} } \right)} \right]} & {\left( {1 - u} \right)\omega_{v} \le \left| \omega \right| \le \left( {1 + u} \right)\omega_{v} } \\ 0 & {otherwise} \\ \end{array} } \right.$$

7 $$\psi_{v} (\omega ) = \left\{ {\begin{array}{*{20}c} 1 & {\left( {1 + u} \right)\omega_{v} \le \left| \omega \right| \le \left( {1 - u} \right)\omega_{v + 1} } \\ {\cos \left[ {\frac{\pi }{2} \times \Gamma \left( {u,\omega_{v + 1} } \right)} \right]} & {\left( {1 - u} \right)\omega_{v + 1} \le \left| \omega \right| \le \left( {1 + u} \right)\omega_{v + 1} } \\ {\sin \left[ {\frac{\pi }{2} \times \Gamma \left( {u,\omega_{v} } \right)} \right]} & {\left( {1 - u} \right)\omega_{v} \le \left| \omega \right| \le \left( {1 + u} \right)\omega_{v} } \\ 0 & {otherwise} \\ \end{array} } \right.$$

where

8 $$\Gamma \left( {u,\omega_{v} } \right) = \psi \left( {\frac{{\left| \omega \right| - (1 - u)\omega_{v} }}{{2u\omega_{v} }}} \right)$$

and

9 $$\psi (x) = \left\{ {\begin{array}{*{20}c} 0 & {x \le 0} \\ {\psi (x) + \psi (1 - x) = 1} & {0 < x < 1} \\ 1 & {x \ge 1} \\ \end{array} } \right.$$

The FBIMF ranks them from high to low according to their respective energy. The energy of each FBIMF is calculated as follows:

10 $$E_{{FBIMF_{i} }} = \sum\limits_{m = 0}^{M - 1} {\left| {FBIMF_{i} (m)} \right|^{2} } ,i = 1,2, \cdots ,N$$

where $E_{{FBIMF_{i} }}$ represents the energy of FBIMF, *M* represents the length of IMF, and *N* represents the total number of FBIMFs.

**Figure 1:**
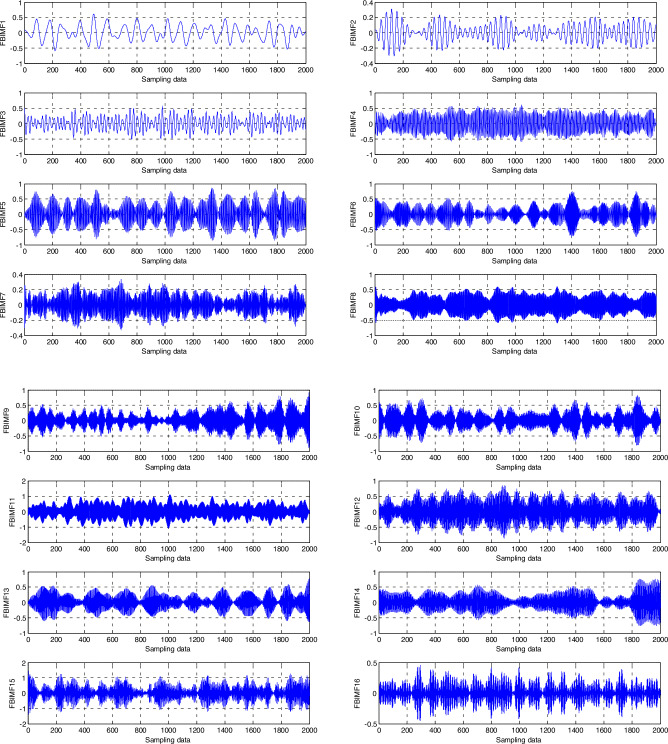
16 FBIMFs of the signal based on FB-EWT.

The FBEWT of each signal results in 16 FBIMFs in this paper, which is shown in Fig. 1.

## Manifold regularization ELM

Most of the existing deep limit learning machines use AE-ELM as the basic module to build a multi-layer network structure to achieve the abstract extraction of data sample features. In order to better maintain the local manifold structure of data in multi-layer unsupervised learning, the ELM with manifold regularization is proposed, where the output of the previous layer is used as the input of the next layer.

11 $$X^{k} = f\left( {\left( {\beta^{k} } \right)^{T} X^{k - 1} } \right)$$

Unlike the supervised learning depth limit learning machine, which uses the traditional ELM as the classifier at the classification level, manifold regularization ELM sends the features extracted by multi-level autoencoders into a semi supervised ELM with regularization constraints. The objective function of the semi supervised ELM is defined as the following formula:

12 $$\min \left\{ {\frac{1}{2}\left\| \beta \right\|^{2} + \frac{\lambda }{2}W\left\| {Z - H\beta } \right\|^{2} + \frac{\mu }{2}Tr\left( {\beta^{T} H^{T} JH\beta } \right)} \right\}$$

where *W* is a diagonal matrix, and the output weight matrix $\beta$ can be obtained by the following formula:

13 $$\beta = \left\{ \begin{gathered} \left( {I_{L} + \lambda H^{T} WH + \mu H^{T} JH} \right)^{ - 1} H^{T} CWZ,n \ge L \hfill \\ H^{T} \left( {I_{n} + \lambda WHH^{T} + \mu JHH^{T} } \right)^{ - 1} CWZ,n < L \hfill \\ \end{gathered} \right.$$

Finally, manifold regularization ELM can be expressed as the following formula:

14 $$F(x) = \left[ {\begin{array}{*{20}c} {K(x,x_{1} )} \\ \vdots \\ {K(x,x_{L} )} \\ \end{array} } \right]\left( {\frac{1}{\xi } + \Omega_{KELM} } \right)^{ - 1} Z$$

where $\xi$ is the regularization coefficient, and $\Omega_{KELM}$ is the kernel function:

15 $$\Omega_{KELM} = \varphi (x_{i} ) \cdot \varphi (x_{j} ) = \exp \left( { - \delta \left\| {x_{i} - x_{j} } \right\|^{2} } \right)$$

## Experimental study and results

The framework of fault diagnosis of gearbox includes motor, gearbox, coupling, load, sensor and acquisition card, and computer. The faults of gearbox include wear and tear, snaggletooth and tooth pitting.

In order to outstand the superiority and stability of FBEWT-MRELM, the diagnosis methods including FBEWT-ELM, EWT-MRELM, and EWT-ELM are respectively used to compare with FBEWT-MRELM under the conditions of the balanced dataset and unbalanced dataset.

**Figure 2:**
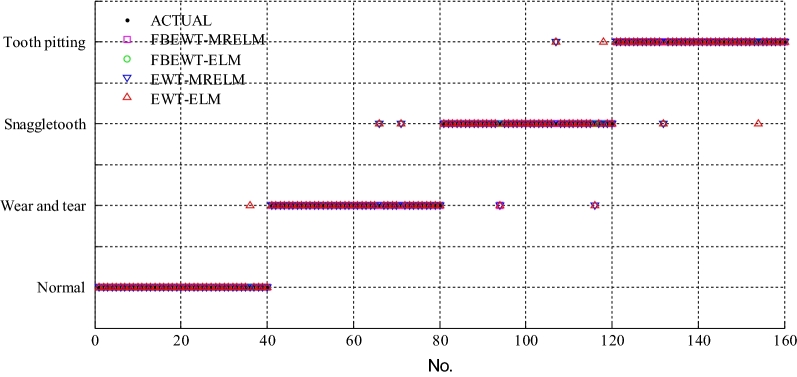
The comparison of diagnosis results of gearbox among FBEWT-MRELM, FBEWT-ELM, EWT-MRELM, and EWT-ELM under the conditions of balanced dataset.

**Figure 3:**
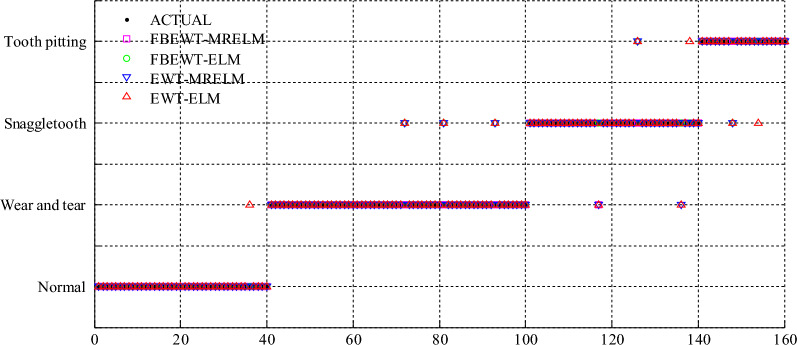
The comparison of diagnosis results of gearbox among FBEWT-MRELM, FBEWT-ELM, EWT-MRELM, and EWT-ELM under the conditions of unbalanced dataset.

Firstly, the balanced dataset is used to testify the superiority of the proposed FBEWT and manifold regularization ELM. The training samples includes 60 samples of each state, and total training samples are 240. The testing samples includes 40 samples of each state, and total training samples are 160. The training samples and testing samples are no intersection. The experimental results are identical and repeatable by some repeated experiments. The comparison of diagnosis results of gearbox among FBEWT-MRELM, FBEWT-ELM, EWT-MRELM, and EWT-ELM under the conditions of balanced dataset are shown in Fig. 2. As shown in Figs. 2  and 3, solid circle represents the real categories of these samples. Other different symbols represent the corresponding diagnosis categories by using the different methods. The overlapping of solid circle and other different symbols indicates that the diagnosis results of the corresponding methods are correct; conversely, the diagnosis results of the corresponding methods are incorrect. As shown in Table 1, the diagnosis accuracy of FBEWT-MRELM of gearbox is 99.375%, the diagnosis accuracy of FBEWT-ELM is 97.5%, the diagnosis accuracy of EWT-MRELM of gearbox is 96.25%, and the diagnosis accuracy of EWT-ELM of gearbox is 94.375%. The experimental results indicate that the diagnosis accuracy of FBEWT-MRELM of gearbox is higher than that of FBEWT-ELM, EWT-MRELM, and EWT-ELM under the conditions of balanced dataset.

**Table 1:** The comparison of diagnosis accuracies among FBEWT-MRELM, FBEWT-ELM, EWT-MRELM, and EWT-ELM under the conditions of balanced dataset.

Method	The total number of testing samples	The number of corrected diagnosis	Accuracy (%)
FBEWT-MRELM	160	159	99.375
FBEWT-ELM	160	156	97.5
EWT-MRELM	160	154	96.25
EWT-ELM	160	151	94.375

Then, the unbalanced dataset is used to testify the superiority of the proposed FBEWT and manifold regularization ELM. The training samples includes 60 samples with normal state, 80 samples with wear and tear, 60 samples with snaggletooth, and 40 samples with tooth pitting, and total training samples are 200. The testing samples includes 40 samples with normal state, 60 samples with wear and tear, 40 samples with snaggletooth, and 20 samples with tooth pitting, and total training samples are 160. The training samples and testing samples are no intersection. The experimental results are identical and repeatable by some repeated experiments. The comparison of diagnosis results of gearbox among FBEWT-MRELM, FBEWT-ELM, EWT-MRELM, and EWT-ELM under the conditions of unbalanced dataset are shown in Fig. 3. As shown in Table 2, the diagnosis accuracy of FBEWT-MRELM of gearbox is 99.375%, the diagnosis accuracy of FBEWT-ELM is 96.875%, the diagnosis accuracy of EWT-MRELM of gearbox is 95.625%, and the diagnosis accuracy of EWT-ELM of gearbox is 93.75%. The experimental results indicate that the diagnosis accuracy of FBEWT-MRELM of gearbox is higher than that of FBEWT-ELM, EWT-MRELM, and EWT-ELM under the conditions of unbalanced dataset.

**Table 2:** The comparison of diagnosis accuracies among FBEWT-MRELM, FBEWT-ELM, EWT-MRELM, and EWT-ELM under the conditions of unbalanced dataset.

Method	The total number of testing samples	The number of corrected diagnosis	Accuracy (%)
FBEWT-MRELM	160	159	99.375
FBEWT-ELM	160	155	96.875
EWT-MRELM	160	153	95.625
EWT-ELM	160	150	93.75

The experimental results testify that FBEWT-MRELM are more superior and stable than FBEWT-ELM, EWT-MRELM, and EWT-ELM regardless of balanced dataset and unbalanced dataset.

## Conclusions

In order to obtain the excellent fault diagnosis results of gearbox, the novel fault diagnosis method of gearbox based on FBEWT and manifold regularization ELM is proposed to obtain excellent fault diagnosis results of gearbox in this paper. The contributions and novelties of this paper are described as follows: (1) A new feature extraction strategy based on Bessel series expansion-based empirical wavelet transform is used to capture the key non-stationary features of the vibrational signal of gearbox, and significantly improve the diagnosis ability of gearbox; (2) The ELM with manifold regularization is proposed for fault diagnosis of gearbox, and manifold regularization ELM sends the features extracted by multi-level autoencoders into a semi supervised ELM with regularization constraints. The balanced dataset and unbalanced dataset, respectively, are used to outstand the superiority and stability of the proposed FBEWT and manifold regularization ELM. The experimental results testify that FBEWT-MRELM are more superior and stable than FBEWT-ELM, EWT-MRELM, and EWT-ELM regardless of balanced dataset and unbalanced dataset.

## Data Availability

The datasets analyzed during the current study are available from the corresponding author on reasonable request.
